# Coexisting good neighbours: acoustic and calling microhabitat niche partitioning in two elusive syntopic species of balloon frogs, *Uperodon systoma* and *U. globulosus* (Anura: Microhylidae) and potential of individual vocal signatures

**DOI:** 10.1186/s40850-022-00132-x

**Published:** 2022-05-27

**Authors:** Vishal Kumar Prasad, Ming-Feng Chuang, Abhijit Das, K. Ramesh, Yoonjung Yi, K. P. Dinesh, Amaël Borzée

**Affiliations:** 1 Laboratory of Animal Behaviour and Conservation, College of Biology and the Environment, Nanjing Forestry University, 159 Longpan Rd, Xuanwu, Nanjing, 210037 Jiangsu China; 2 Wildlife Institute of India, P. O. Box 18, Dehradun, Uttarakhand 248001 India; 3 Department of Life Sciences, National Chung Hsing University, Taichung, Taiwan; 4 Division of EcoScience, Ewha Womans University, 52, Ewhayeodae-gil, Seodaemun-gu, Seoul, 03760 Republic of Korea; 5 Zoological Survey of India, Western Regional Centre, Pune, Maharashtra 411044 India

**Keywords:** Acoustic segregation — microhabitat partitioning — assortative mating — individual vocal distinctiveness— sympatric species — syntopic species — vocal behaviour — behavioural ecology

## Abstract

**Background:**

Most amphibians use a repertoire of acoustic signals to propagate signals in social contexts. The description of these repertoires provides a key towards the understanding of the behaviour of individuals and the evolutionary functions of calls. Here, we assessed the variations in advertisement calls within and between two fossorial sympatric species, *Uperodon systoma* and *Uperodon globulosus*, that share their breeding season and breeding sites. For each species, we applied Beecher’s index of total information capacity (H_S_) for the individual vocal signature, determined the difference in call properties and demonstrated the segregation in the calling microhabitat niche between the two species.

**Results:**

Our results demonstrated that the advertisement calls of *U. systoma* are pulsatile with a call rate of 3.00 ± 0.97 calls per second while those of *U. globulosus* are not pulsatile with a lower call rate of 0.53 ± 0.22 calls per second. For both species, the variations in call properties among individuals was higher than that within individual, a pattern consistent with that of other fossorial anurans. The body condition and air temperature did not significantly impact the call properties of either species. The outcome of the Beecher’s index (H_S_) showed that the calls of *U. systoma* can be used to identify 14 different individuals and the calls of *U. globulosus* can be used to identify 26 different individuals. The statistical analyses on the advertisement call of the two species showed a significant difference in the temporal properties as the call duration, and fall time and rise time were significantly different between the two species. Lastly, we successfully demonstrated that there is a clear segregation in calling site microhabitat between the two species, where *U. globulosus* calls floating close to the bank of the waterbody while *U. systoma* calls floating further away from the bank.

**Conclusion:**

This study highlights the potential for pre-mating isolation, character displacement and assortative mating in two syntopic fossorial anurans, leading to association between acoustic, calling microhabitat niche and body index divergence as important behavioural and ecological traits.

**Supplementary Information:**

The online version contains supplementary material available at 10.1186/s40850-022-00132-x.

## Background

Syntopic species temporally co-occur in the same habitat and they generally compete for resources, often resulting in habitat segregation and distinct vocal behaviours [1–3]. During the breeding season, anuran species produce vocal signals that help provide vital information about territory [4, 5], mate attraction [6], female mate choice [7], competition between males [8], fighting ability [9], body size of opponent during aggressive encounters [10, 11], and species identity [12, 13]. Vocal signals are distinctive for every species and are also used for non-territorial scramble competition, where males outnumber females in breeding aggregations that last for a short period [3], lekking where males perform courtship display [14], and for the defence of territories that contain breeding sites [3]. Comprehensive knowledge of a species vocalisation is thus essential to understand its behaviour. For example, calling individuals are less aggressive towards neighbours than stranger individuals in *Babina adenopleura* [5].

Distinct amphibian species may be forced to utilise the same calling habitat when under limited resources, and this may breach pre-mating isolation barriers based on species recognition [1, 5]. As a result, the risk of interspecific mating increases, leading to unsuccessful reproduction or hybridisation [1, 6]. To overcome this problem, species follow non-exclusive strategies to exploit segregated habitat niches: spatial, temporal and acoustic partitioning and preferences [3, 15–19]. In complex and resource scarce environments where several species are under pressure to breed at same time, differences in advertisement calls enable species-specific recognition of acoustic signals [20]. As a result, syntopic species using similar breeding habitats can remain isolated through differing acoustic properties. For example, Robber frog species (*Eleutherodactylus* spp.) from Puerto Rico calls during the same diel period but exhibits partitioning in the call frequency [18]. Another example is that of the American bullfrogs (*Lithobates catesbeiana*) and Green frogs (*Lithobates clamitans*; taxonomy following Frost 2021 [21];) occupying similar breeding ponds and sharing the same breeding season and diel period. The calls of the earlier species last longer and are more frequent than that of the later species, and to avoid acoustic interference *L. clamitans* adapts to place its calls in silent gaps between the calls of *L. catesbeianus* [16].

Environmental variables are crucial in influencing the behaviour of a species towards the selection of appropriate breeding sites [22]. For instance, the temperature can affect the call properties of anurans, and the air and water temperatures of calling sites positively influence the calling activity in *Pseudacris crucifer* [23]. Comparably, *Pelophylax nigromaculatus* calls at higher relative humidity than the sympatric *Rana dybowskii,* resulting in temporal partitioning [24]. Similarly, the relative humidity also indicates the end of the breeding period in *Dryophytes suweonensis* [22]. Hence, understanding the environmental variables associated with the advertisement calls of amphibians greatly help understand their behaviour.

With over 8000 described anuran species in the world [21], there is still very limited knowledge available about the vocal behaviour of some of the species. India harbours 413 anuran species [25], but there have been only few studies of bioacoustics of anurans [26–33]. To date, there is no detailed study on the vocal behavior of any species in the genus *Uperodon*. *Uperodon systoma* and *U. globulosus* share a large portion of their ranges and also have the same breeding season and sites. Thus, the spatial and acoustic partitioning are expected in these two species. Here, we provide: 1) a description of the advertisement calls of two elusive Balloon frog species (*Uperodon systoma* and *Uperodon globulosus*) from central India; 2) differences in advertisement call properties between the two species; 3) the individual signal signature recognition for each species; 4) microhabitat segregation at calling sites between the two species.

## Results

### Advertisement call of *Uperodon systoma*

**Fig. 1:**
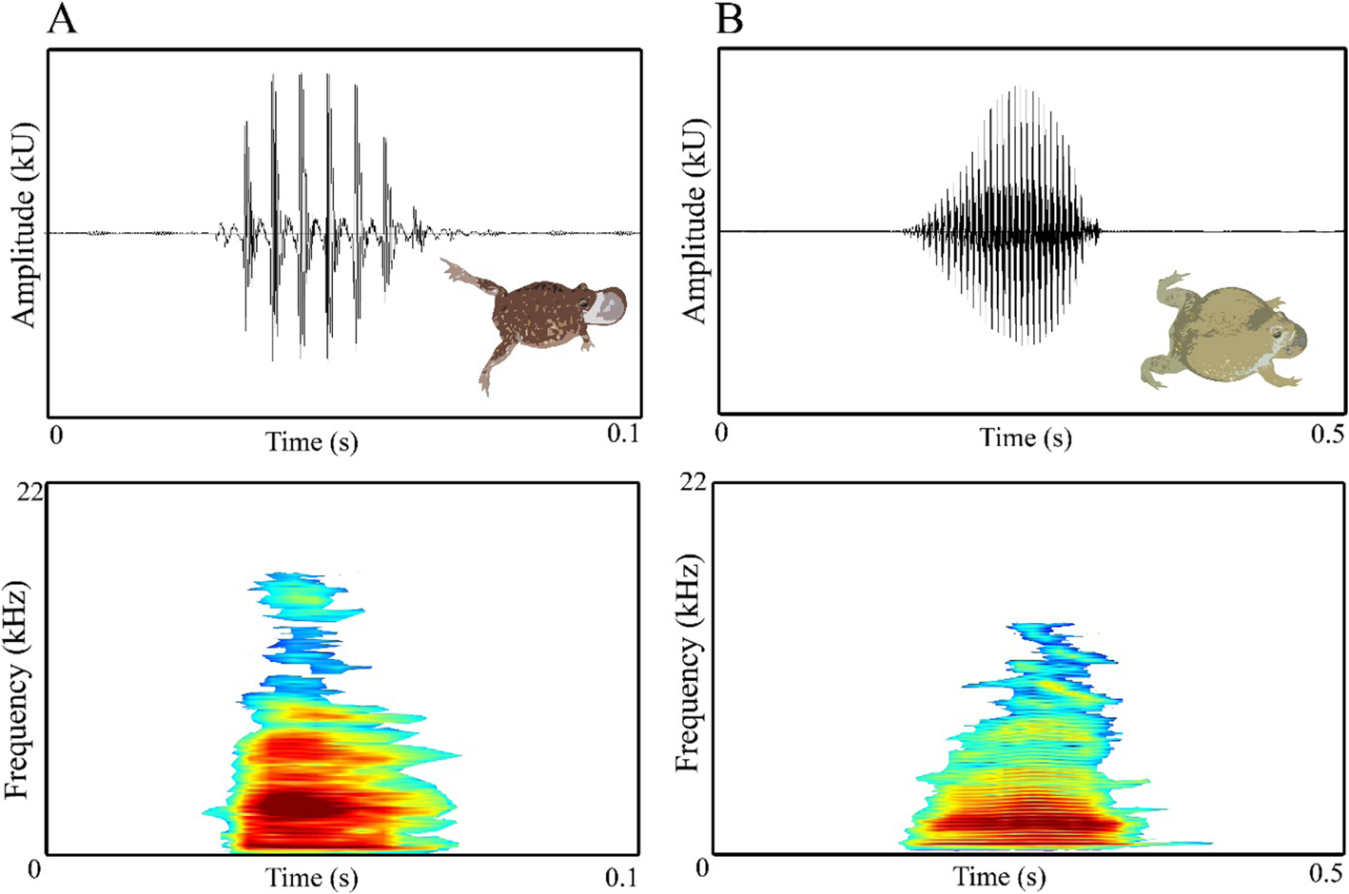
Schematic illustrations of vocal repertoire of the two Balloon frog species: (**A**) *Uperodon systoma* and (**B**) *Uperodon globulosus*. Here, the call property is depicted in an oscillogram and a spectrogram (FFT size = 1024 pts., Hanning window, 43.1 Hz resolution)

The advertisement call (Fig. 1A) of *Uperodon systoma* is quick and densely pulsed, typically consisting of an average of 6–7 consecutive pulses. The call duration is short (38.73 ± 4.52 ms) and the call rate is high (3.00 ± 0.97/s) with approximately 12 harmonics with power (sidebands) present in each call (Table 1). A second emphasized harmonic is present in the advertisement calls of *U. systoma*, and the dominant frequency is the high frequency in this species. The results of the ANOVA tests for variation in calls among and within individuals of *Uperodon systoma* demonstrate that the coefficient of variation (CV) of the temporal and spectral call properties is more variable among individuals (CV_a_) than within individuals (CV_w_) for all the properties except for frequency modulation of the low frequency (Table 1). The coefficient of variation among individuals (CVa) was calculated by dividing the standard deviation value of all individuals by the average value of all individuals (CVa = SD/Mean value, all individuals). This variation is reflected in the CV_a_:CV_w_ ratios which ranged between 1.01 to 2.60, with the exception of frequency modulation of low frequency (0.27; Table 1). The CV_a_:CV_w_ ratio is higher (2.6) for low peak frequency than for all other call properties. The result of the correlation test to check for relationship between call properties and other variables showed that the coefficient of correlation with air temperature and body condition is not significant for any of the call properties (Table S1). The result of the model II ANOVA shows that call properties were significantly different among individuals (Table 1).

**Table 1:** Exploratory data analysis of the calls of *Uperodon systoma*

Call property	Mean	Range	CVw (%)	CVa (%)	CVa/CVw	F_12, 354_	*P*	η2
Call rate (1/s)	3 ± 0.97	0.22–5.98	26.53^d^	26.99	1.017	17.67	**< 0.001**	0.466
Call duration (ms)	38.73 ± 4.52	30–57	6.67^i^	9.71	1.455	167.14	**< 0.001**	0.912
Rise time (ms)	19.41 ± 2.27	15–28	6.79^i^	10.4	1.532	165.79	**< 0.001**	0.911
Fall time (ms)	19.41 ± 2.28	15–28	7.02^i^	9.25	1.318	172.11	**< 0.001**	0.914
Low peak frequency (Hz)	443.98 ± 18.82	408–491.4	1.65^s^	4.3	2.605	120.53	**< 0.001**	0.850
High peak frequency (Hz)	2689.53 ± 162.71	2290.3–3147.5	2.92^s^	5.95	2.033	51.92	**< 0.001**	0.709
Delta power (dB)	7.76 ± 4.08	− 0.4 – 19.7	29.52^d^	48.44	1.641	36.49	**< 0.001**	0.631
FM of low frequency (Hz)	−16.99 ± 17.66	−66 – 27.8	−109.74^d^	−30.17	0.275	2.09	**< 0.05**	0.089
FM of high frequency (Hz)	−173.43 ± 150.87	− 559.6 – 250.4	−52.95^d^	−76.13	1.438	15.86	**< 0.001**	0.426
Dominant harmonic	12.14 ± 0.88	9.88–14.21	3.34^s^	6.7	2.005	67.63	**< 0.001**	0.760

The table shows the values of coefficient of variation for 10 properties of advertisement calls of *Uperodon systoma* measured within individuals (CVw) and among individuals (CVa), the ratio of among–individual to within–individual variation (CVa/CVw), and results from model II ANOVAs (all Ps <  0.001 or 0.05). “FM” = frequency modulation; “CVw” = Coefficient of variation within individuals; “CVa” = Coefficient of variation among individuals; “d” = dynamic call; “s” = static call; “I” = intermediate call

### Advertisement call of *Uperodon globulosus*

The advertisement calls (Fig. 1B) are long and not pulsatile. The call rate is low (0.53 ± 0.22/s) and call duration is long (154.28 ± 23.11 ms). The second emphasized harmonic is not present in the advertisement calls of *U. globulosus* unlike *U. systoma*. The results of the ANOVA testing for the variation among and within the calls of *U. globulosus* show that the coefficient of variation (CV) of the temporal and spectral call properties is more variable among individuals (CV_a_) than within individuals (CV_w_) for all call properties except for call rate and frequency modulation of low frequency (Table 2). This variation is reflected in the CV_a_:CV_w_ ratios, which ranged between 3.09 and 10.67, except for call rate (0.60) and frequency modulation of low frequency (Table 2). Among all call properties, the CV_a_:CV_w_ ratio is the highest for low peak frequency (10.67) followed by frequency modulation of high frequency (9.67) and delta power (5.72). Additionally, the outcome of the correlation test to determine the relationship between call properties and environmental variables showed that the coefficient of correlation was not significant for any of the call properties with air temperature and body condition (Table S1). The result of model II ANOVA further reveals that all of the call properties were significantly different among individuals (Table 2).

**Table 2:** Exploratory data analysis of the calls of *Uperodon globulosus*

Call property	Mean	Range	CVw (%)	CVa (%)	CVa/CVw	F_6, 250_	*P*	η2
Call rate (1/s)	0.53 ± 0.22	0.05–1.23	33.244	19.990	0.601	4.10	**< 0.05**	0.215
Call duration (ms)	154.28 ± 23.11	108–198	4.367	16.765	3.839	167.14	**< 0.001**	0.912
Rise time (ms)	77.16 ± 11.53	54–99	4.357	16.707	3.835	165.79	**< 0.001**	0.911
Fall time (ms)	77.15 ± 11.52	54–99	4.253	16.626	3.909	172.11	**< 0.001**	0.914
Low peak frequency (Hz)	432.82 ± 45.91	392.1–587.3	1.206	12.873	10.673	1067.60	**< 0.001**	0.985
High peak frequency (Hz)	1517.51 ± 331.36	618.2–2040.1	7.009	21.641	3.088	59.88	**< 0.001**	0.787
Delta power (dB)	2.86 ± 2.87	−9.2 – 6.2	12.989	74.342	5.723	19.95	**< 0.001**	0.552
FM of low frequency (Hz)	10.91 ± 38.77	−164.9 – 113.3	–	202.932	0.000	10.49	**< 0.001**	0.393
FM of high frequency (Hz)	−45.5 ± 546.68	− 3071.3 – 1876.1	−20.731	−200.388	9.666	5.40	**< 0.001**	0.250
Dominant harmonic	7.18 ± 1.91	2.93–10.22	6.936	28.365	4.090	102.28	**< 0.001**	0.864

In the table, the coefficient of variation for 10 properties of advertisement calls of *Uperodon globulosus* measured within individuals (CVw) and among individuals (CVa), the ratio of among-individual to within-individual variation (CVa/CVw), and results from model II ANOVAs (all Ps < 0.001 or < 0.05)

### Individual vocal distinctiveness

To show the individual vocal signature, we statistically calculated the variation in the ten call properties we measured for each species. In the case of *U. systoma*, our result of the model II ANOVA shows that the values of partial η2, that represent the size of the effect of individual identity, ranged between 0.09 and 0.91 (Table 1). Regarding the spectral properties, the low peak frequency has the highest value of partial η2 (0.85) and the greatest CVa/CVw ratios (mean of 2.60; Table 1). This is the lowest variation within individuals with a mean CVw value of 1.7%. The dominant harmonic and low peak frequency also have high values for partial η2 (means of 0.76 and 0.85 respectively) and also high values of CVa/CVw ratios (means of 2 and 2.60 respectively). With CVw values of − 1.097 and − 0.53% respectively, the frequency modulation for the low frequency and frequency modulation for the high frequency are most variable within the individuals compared to other spectral properties. The values for the coefficients of variation related to the high and low frequency modulations are negative because frequency modulations were calculated by subtracting the value of the low frequency from the last 12 ms window of power spectrum of the call and the value of low frequency for the first 12 ms window of power spectrum of the call measured. The value of the low frequency for the first 12 ms was greater than that of the low frequency for the last 12 ms, similarly to the high frequency. This resulted in negative values of frequency modulations for both low frequency and high frequencies. In temporal properties, call fall time and call duration (Table 1) have the highest values of partial η2 (mean of 0.91 and 0.91 respectively). Importantly, the total information capacity (H_S_) of *U. systoma* advertisement calls is 3.83 bits, signifying that these signals can uniquely identify an upper limit of ~ 14 (value: 14.25) different individuals, assuming an ideal receiver [34].

In the case of *Uperodon globulosus,* our result of the model II ANOVA shows that the values of partial η2, ranged between 0.21 and 0.98. In the spectral properties, the low peak frequency has the highest value of partial η2 (0.98) and the greatest CVa/CVw ratios (mean of 10.67; Table 2). This spectral property also displays the lowest variation within individuals with a mean CVw value 1.2% and also the lowest variation among individuals with a mean CVa value 12.9%. Dominant harmonic and high peak frequency also have high partial η2 values (means of 0.86 and 0.79 respectively) and also high values of CVa/CVw ratios (means of 4.90 and 3.09 respectively). Regarding the temporal properties, fall time, call duration and rise time (Table 2) have the highest partial η2 values (mean of 0.91 each respectively) and CVa/CVw ratios values, 3.86, 3.81 and 3.71 respectively (Table 2). The total information capacity (H_S_) of *U. globulosus* advertisement calls is 4.69 bits, signifying that these signals can distinctively identify an upper limit of 26 (value: 25.85) different individuals, assuming an ideal receiver [34].

### Determining difference in advertisement calls between two syntopic species

**Fig. 2:**
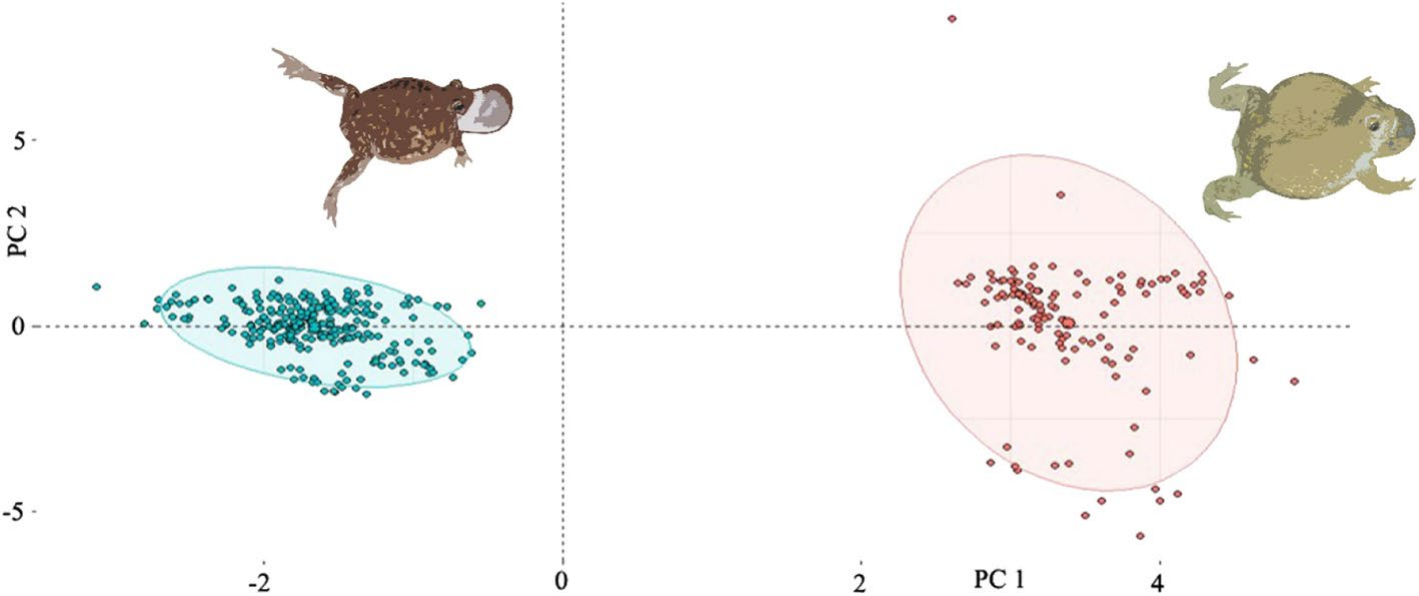
Result of the Principal component analyses of ten acoustic variables showing partitioning in the call properties between *Uperodon systoma* (green confidence ellipse) and *U. globulosus* (red confidence ellipse) resulting from PC1 (consisting mostly temporal properties) and PC2 (consisting mostly spectral properties of calls). The PCA is based on 257 of *U. systoma* (*n* = 13) and 111 calls of *U. globulosus* (*n* = 7)

The Principal Component Analysis (PCA) to identify the difference in call properties between *U. systoma* (256 calls from 13 individuals) and *U. globulosus* (111 calls from 7 individuals) results in two PCs, with eigenvalues values of 5.94 and 1.63, explaining a cumulated variance of 75.62% (Table 3). The PC1 axis explains 59.35% of the total variation, mostly related to temporal properties as it includes call duration, call fall time and call rise time. The PC2 axis explains 16.27% of the variation, mostly through spectral properties. The result of the ANOVA to determine variation in call properties between the two species is significant for PC1 (*p* = 0.001, df = 1, *F*_*df1, df2*_ = 11,414), but not for PC2 (*p* = 0.367, df = 1, *F*_*df1, df2*_ = 0.816). The confidence ellipses for each PCs do not overlap (Fig. 2), highlighting the acoustic segregation in the call properties of the two species of *Uperodon*.

**Table 3:** Principal components and their values resulting from the PCA computed to segregate acoustic properties between the two species

Principal components	PC1	PC2
Call Rate	−0.34	−0.09
Call Duration	**0.40**	0.06
Rise Time	**0.40**	0.06
Fall Time	**0.40**	0.06
Low Frequency	−0.03	**−0.71**
High Frequency	−0.39	−0.04
Delta Power	−0.24	−0.03
Frequency Modulation Low Frequency	0.22	**−0.37**
Frequency Modulation High Frequency	0.09	−**0.58**
Dominant Harmonic	−0.38	0.04
Standard Deviation	2.44	1.28
Percentage of Variance	59.35	16.27
Eigenvalues	5.94	1.63
*ANOVA*		
*p*	0.001	0.367
Df	1	1
*F*_*df1, df2*_	11,414	0.816

In bold are variables retained as loading into one of the PCs, and significant *p*-values from the statistical analysis to test for differences between species. The PCA is based on 257 calls of *U. systoma* (*n* = 13) and 111 calls of *U. globulosus* (*n* = 7)

### Ecological requirements and habitat segregation

**Fig. 3:**
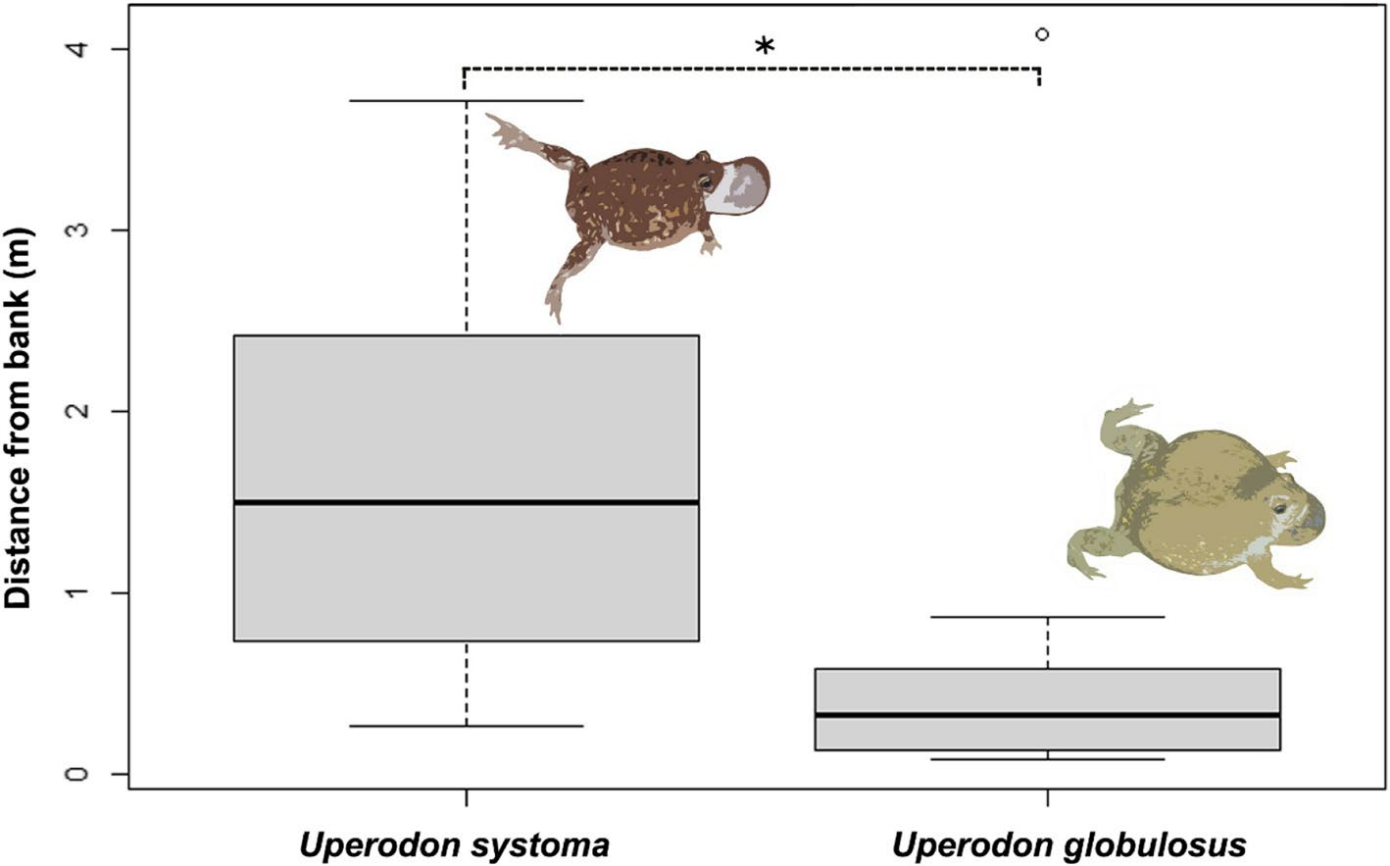
Distributions of distance from bank for *Uperodon. systoma* (*n* = 15) and *U. globulosus* (*n* = 16) in puddles and pool. At night, the calling males of the two species of *Uperodon* produced advertisement calls, and they distributed themselves in the puddles and pool. Here “Distance from bank” was the distance between the bank and the calling male’s location floating in the puddle

To determine niche segregation in calling microhabitats, we compared the calling location of males of the two *Uperodon* species. The result of the one-way ANOVA shows that when calling syntopically, the distance to the bank is significantly different between the males of two species (Kruskal-Wallis chi-squared = 15.95, *n* = 31, *p* = 0.001, Table 4, Fig. 3). *Uperodon systoma* calling males (*n* = 15) are located 1.64 ± 1.10 m (mean ± sd) from the bank, while *U. globulosus* calling males (*n* = 16) are 0.58 ± 0.96 m from bank of water bodies (Fig. 3). The two species are also significantly different in body condition index (Kruskal-Wallis chi-squared = 4.84, *n* = 31, *p* = 0.028), with *U. globulosus* having a higher body condition and being larger 53.57 ± 2.20 mm (mean ± sd) and heavier 20.65 ± 4.36 g than *U. systoma* (52.15 ± 2.49 mm and 17.99 ± 5.01 g).

**Table 4:** Representing the results of one-way ANOVA test of body index and environment variables

Variables	Kruskal-Wallis chi-squared	df	*p*-value
Body index (mm/g)	4.844	1.00	**0.028**
Air temp (°c)	1.026	1.00	0.311
Water temp (°c)	0.431	1.00	0.512
Relative humidity (%)	0.509	1.00	0.475
Wind speed (km)	3.256	1.00	0.071
Depth (m)	2.206	1.00	0.138
Distance/Area (m)	15.952	1.00	**0.001**

These variables are of calling sites for significant difference between the *Uperodon systoma* and *Uperodon globulosus*. Significant values are in bold

Our result of the one-way ANOVA to compare variation in wind speed between the two species when they produced advertisement calls is close to significance (Kruskal-Wallis chi-squared = 3.25, *p* = 0.071; *n* = 31; Table 4). *Uperodon systoma* produces advertisement calls when the wind speed is almost zero (0.09 ± 0.24 km/hour (mean ± sd), *n* = 14) and *U. globulosus* produces advertisement calls even when the wind speed is relatively higher (1.79 ± 2.04 km/hour, *n* = 16). The other environmental variables: air temperature, water temperature, relative humidity and depth of water are not significantly different between the two species (Table 4).

## Discussion

We determined that the two species, *Uperodon systoma* and *U. globulosus*, are significantly different in terms of call properties, individual call distinctiveness and microhabitat use when producing advertisement calls. The drivers of adaptive divergence in call properties and microhabitat use for *Uperodon* are not known, but our results suggest that they may be related to prezygotic isolation [35] and character displacement [36], similarly to numerous other species such as Little greenbul [37], Flour beetles [38] and Green tree frogs [39]. Character displacement lessens the competition for breeding resources in sympatric species by promoting the segregation of resource use and habitat related phenotypes. However, to ascertain the presence of character displacements in *Uperodon*, a comparison with population in allopatry would be needed.

In addition, the distinctiveness in advertisement call properties between the two *Uperodon* species is likely related to mate choice, with females able to distinguish signal clearly, an important requirement for explosive breeders breeding in syntopy within limited resources [7, 16]. For example, females of syntopic cricket frogs (*Acris* spp.) preferred fine temporal structure to discriminate between conspecific and heterospecific vocalizations, and body size had a more important effect on the dominant frequency of *Acris crepitans* than *A. gryllus* [40]. In contrast, advertisement calls may not be the most important trait to attract females as being explosive breeders, *U. systoma* and *U. globulosus* also rely on scramble competition. This breeding strategy relies on calls to attract mates but may not need to advertise the fighting potential of males once in the spawning area, in opposition with resource defence breeders. For example, the female Columbia spotted frog (*Rana luteiventris*) selects males with greater sexual dimorphism such as enlarged nuptial pads and thicker forearms [41], and in Moor frogs (*Rana arvalis*), a larger size increases mating success during scramble competition [42]. The difference in the body index between the two *Uperodon* species may reflect additional differences in behaviours, such as size-assortative mating during scramble competition [43, 44].

Most call properties are significantly different among individuals in both of the focal *Uperodon* species, highlighting a potential to identify individual by their calls. The total information capacity [34] for both *Uperodon systoma* (3.83 bits; ~ 14 individuals distinguishable) and *U. globulosus* (4.69 bits; ~ 26 individuals distinguishable) are relatively low compared to other anurans species such as *Nidirana adenopleura* (7.3 bits; ~ 158 distinguishable individuals [5];) and birds such as the Lazuli buntings (*Passerina amoena*; 10.6 bits; ~ 1552 distinguishable individuals [45];). This difference in information capacity between the two *Uperodon* species may result from a preference for breeding in small rainwater puddles which cannot accommodate a large number of calling individuals due to their small size. We did not observe more than seven *U. systoma* individuals and maximum of six individuals of *U. globulosus* in a single puddle in the field.

Our values of total information capacity are calculated assuming ideal conditions for receiver i.e. females or conspecific males and the value of total information capacity exceeds the typical number of calling neighbours in the field. The total information capacity in nearly more than the double of the actual number of frogs calling from the same water body in the field. Under natural conditions, signals are degraded due to propagation in the environment [46, 47], the ambient noise also masks important signals [48] and receivers can also see they signal degraded in rainy nights or in the presence of other simultaneously calling species during breeding period. We provide values of total information capacity in a broader context because detailed studies would be needed to answer specific questions. However, studies similar to parent offspring recognition in swallows are highly difficult with frogs as there are no estimates of individual identity information for other species of frogs [34, 49].

The two sympatric *Uperodon* species use the same habitat but differ in their call properties (noting that the difference in variation could be linked to sample size; Fig. 2) and microhabitat use (Fig. 3). This may mean that these sympatric species undergo prezygotic isolation to avoid hybridisation through a combination of variations in call properties and microhabitat use. The difference in calling microhabitat may also represent an adaptive response to interspecific competition where *U. globulosus* is dominant as it calls near the bank and *U. systoma* calls away from bank of waterbodies. For instance, treefrogs follow this pattern, competing over calling sites with the dominant *Dryophytes japonicus* calling from the edge of paddy field and the rare *D. suweonensis* calling from the interior of rice paddies [50]. In addition, *U. systoma* calls at lower wind speed compared to *U. globulosus*, a variable affecting the calls of other species (e.g., *Lithobates catesbeianus* [51];). This difference in behaviour may be related to a difference in tolerance to desiccation between the two species.

## Conclusion

To summarise, our study provides the first detailed analyses on the vocal repertoires of *Uperodon systoma* and *U. globulosus*, demonstrating acoustic partitioning between the two syntopic species. The study successfully demonstrated that there is a potential for individual recognition by individual vocal signatures in both species. The values of the Beecher’s index of total capacity information for both species were moderate compared to other anurans [5] and birds [45], however, this needs further assessment. A clear acoustic and microhabitat niche partitioning between the two syntopic species may be related to pre-zygotic isolation, character displacement and assortative mating strategy in the two species. However, the confirmation of character displacement would need to include allopatric populations to be confirmed. The combination of these ecological modes of segregation led to reproductive divergence and the use of different ecological niches by these two fossorial species. The findings of this study broaden our prospective on how two coexisting species diverge in their behaviour and ecology while breeding in the same habitat, thereby increasing our understanding of selective pressure on sympatric species in resource scarce habitats.

## Material and methods

### Study species

**Fig. 4:**
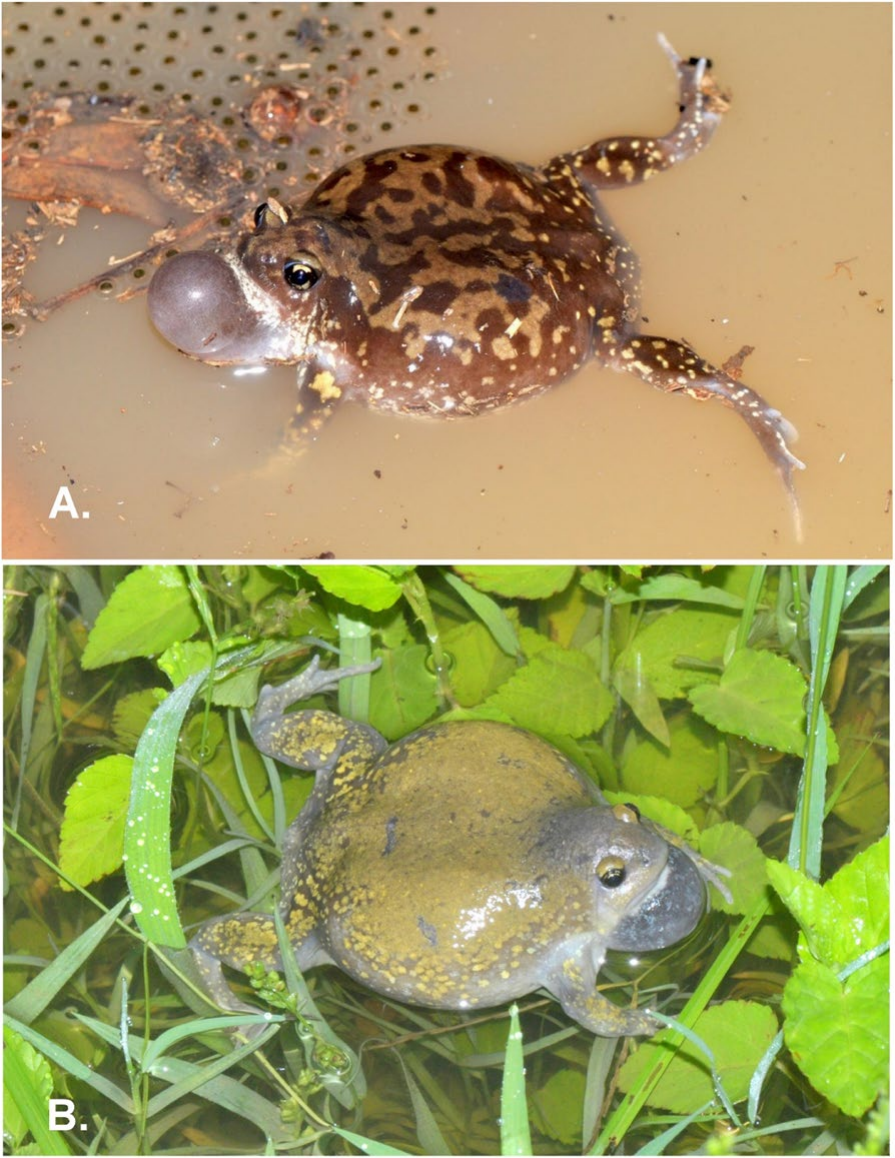
Males of *Uperodon systoma* (**A**) and *U. globulosus* (**B**) in their natural calling habitat producing advertisement calls during rainy season in July at the study site in sub-urban areas in Panna district. Photographs by Vishal Kumar Prasad

The Marbled Balloon Frog (*Uperodon systoma*) is widely distributed in India and Sri Lanka and in the adjoining regions of Pakistan and Nepal. The Indian Balloon Frog (*Uperodon globulosus*) is widely distributed in India and in the neighbouring regions of Bangladesh, Bhutan and Nepal [52]. The two *Uperodon* species occur in sympatry as their distribution range in the Indian subcontinent overlaps [52, 53], although in the central Indian landscape *U. systoma* is rare and *U. globulosus* is uncommon [33]. They surface only for explosive breeding during the pre-monsoonal showers in the months of June and July [52–54]. These two species are highly elusive and fossorial, which makes them difficult to sample [54]. The males of both species produce advertisement calls while floating on the surface of shallow temporary rainwater puddles and pools ([33, 54]; Fig. 4).

### Study site

The calls were recorded in the agricultural fields of Jaruapur (24.70322 N, 80.12527 E), Hinota (24.6494 N, 80.02416 E), and Jangipura (24.73947 N 079.89311 E) villages and in the sub-urban areas in Panna district (24.71467 N, 80.15245 E) of Madhya Pradesh, India. The landscape is characterised by tropical and subtropical dry broadleaf vegetation. The calls were heard from about 19:00 hours up to 04:00 hours the next day strictly after episodes of heavy rains. All waterbodies used for data collection were independent, located 0.20 km to 12 km of each other.

### Data collection

#### Acoustic recordings

We recorded the advertisement calls of *U. systoma* between 01 and 07 July 2019 and calls of *U. globulosus* between 30 June and 26 August 2019 (recordings deposited at FonoZoo sound library [http://www.fonozoo.com/fnz_buscar.php] under accession numbers: 14171 —14,194). We used a unidirectional handheld microphone (Sennheiser MKH 416; Germany) and a digital recorder (Marantz PMD 620 MK–II; China). The input channel of the microphone was handheld approximately 0.5 m away from the pulsating vocal sac of the focal calling animal. The gain settings were adjusted manually before the start of each recording and were kept constant during call recording. Calls were recorded at a sampling rate of 44.1 kHz with 16-bit resolution. After completing each recording, we captured the focal calling male and measured its snout-vent length (SVL) to the nearest 0.01 mm using digital calliper (CD-6”CSX, Mitutoyo Corp, Japan) and we recorded the body mass to the nearest to 0.1 g using a spring scale (Pesola Lightline 50 g; Switzerland).

#### Ecological data

We recorded air and water temperature to the nearest to 0.1 °C, relative humidity (%), wind speed (km/hour; EN150 hygro-thermo-anemomenter, Extech; USA), area of water body (m^2^), depth of water body (m), sex of recorded individual, and the number of individuals present at the calling site, date and time after each call recording. We recorded the GPS locations of each waterbody using a GPS MAP 78S (Garmin; USA) following the WGS84 datum.

#### Advertisement calls and individual vocal distinctiveness

**Fig. 5:**
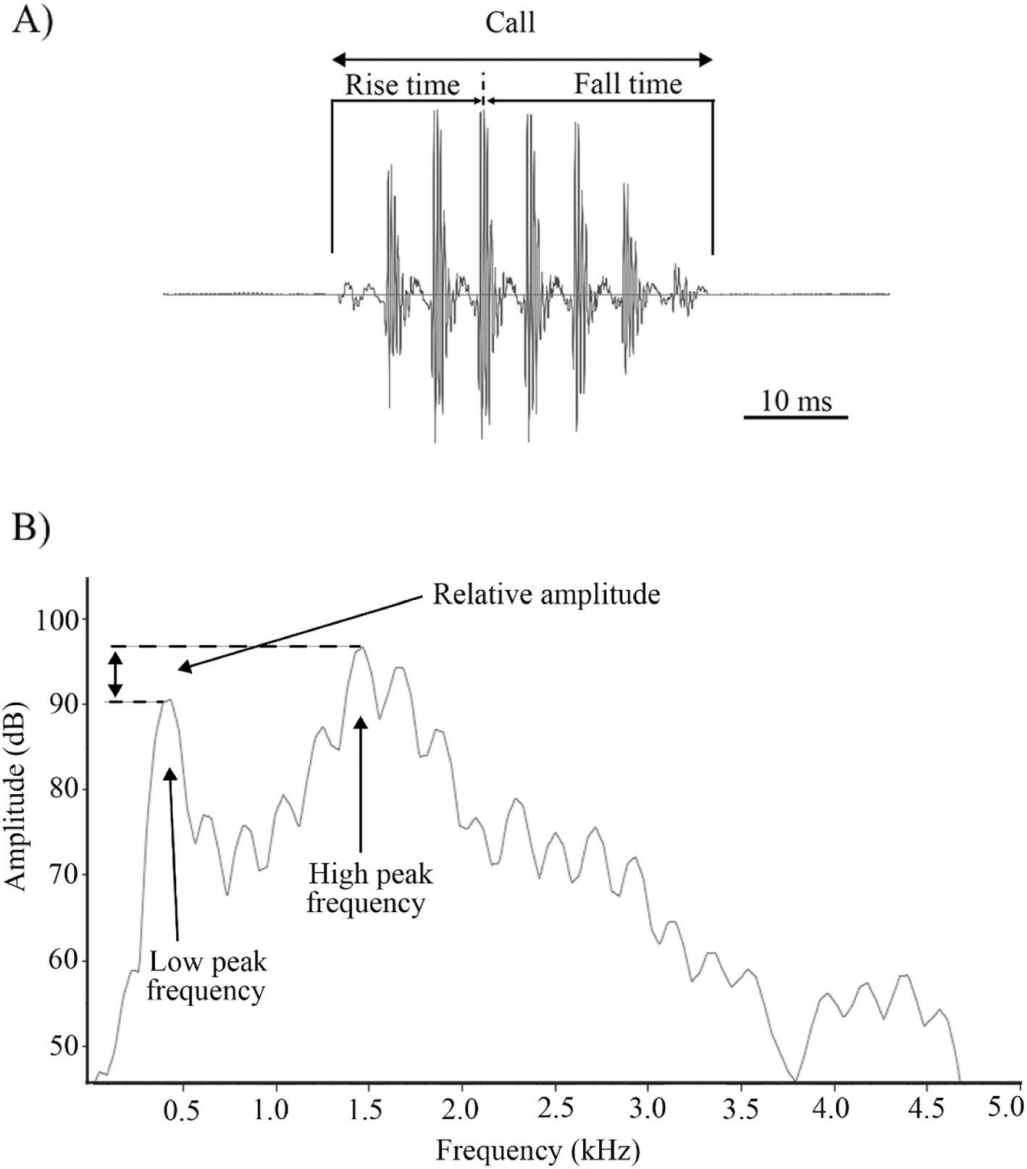
Acoustic measurements. Depicted here are schematic illustrations of measurements of several (**A**) temporal call properties from Raven’s waveform display and (**B**) spectral properties computed from Raven’s power spectrum display (1024 pt. FFT)

We recorded advertisement calls of 13 male *Uperodon systoma* and seven male *U. globulosus*. We extracted call properties based on the recommendations mentioned in the research articles [5, 55, 56]. The ten call properties were: call rate (ms), call duration (ms), rise time (ms), fall time (ms), low frequency (Hz), high frequency (Hz), delta power (dB), frequency modulation of low frequency (Hz), frequency modulation of high frequency (Hz) and dominant harmonic count (terminologies defined in Table 5; Fig. 5). To obtain the frequency modulation of the high frequency and low frequencies, we calculated the low peak frequency and the high peak frequency in the bimodal spectrum, and their relative amplitude, in two separate 12 ms windows (FFT size = 512 pts., Hanning window, 43.1 Hz resolution) using the software Raven Pro 1.5 [57].

**Table 5:** The description of temporal and spectral properties of calls

Call property	Description
Call rate	Number of calls per minute. It is inverse of call period.
Call duration	Duration between start of the first pulse and end of the last pulse of a call
Rise time	Time between start of the first pulse and spike of peak amplitude in the pulse of highest amplitude.
Fall time	Time between spike of peak amplitude in the pulse of highest amplitude and end of the last pulse in a call.
Low peak frequency (Hz)	Maximum frequency in the range of 0.3–0.8 kHz (low peak) determined over the duration of a call (FFT size = 1024 pts., Hanning window, 43.1 Hz resolution).
High peak frequency (Hz)	Maximum frequency in the range of 0.8–3.0 kHz (high peak) determined over the duration of a call FFT size = 1024 pts., Hanning window, 43.1 Hz resolution).
Delta power (dB)	The maximum power in a call
FM of low frequency (Hz)	Difference in the low frequency between last 12 ms and first 12 ms window of the call measured by producing a power spectrum from selection spectrum function (FFT size = 512 sample size, Hanning window, 43.1 Hz resolution) of Raven Pro Bioacoustics Research Program software over the duration of the entire call
FM of high frequency (Hz)	Difference in the high frequency between last 12 ms and first 12 ms window of the call measured by producing a power spectrum from selection spectrum function (FFT size = 512 sample size, Hanning window, 43.1 Hz resolution) of Raven Pro Bioacoustics Research Program software over the duration of the entire call
Dominant harmonic	Energy concentrated in separated and evenly spaced frequency of the wave of the longest wavelength

### Statistical analysis

To nullify the effect of temperature on the acoustic properties, we controlled the values of each variable to the average temperature of all calls by 25.9 °C for *U. systoma* and 24.8 for *U. globulosus*. To do this, we computed the linear equation of each variable in the slope function and calculated the controlled variable at 25.9 °C for *U. systoma* and 24.8 °C for *U. globulosus*. We computed an index of physical condition (i.e. length-independent mass) dividing the measurements of SVL by mass following [58].

To measure the variation in acoustic properties within individuals of the two target species, we tested the hypothesis that advertisement calls are individually distinct [5, 34]. To do so, we performed a model II ANOVA on the ten call properties described above to determine the effect of individual identity and ratio of coefficient of variation among and within individuals (CVa/CVw). We also utilised the Beecher’s information statistic (H_S_) to determine the individual distinctiveness or signal signature in the advertisement calls [34].

To test for significant differences in call properties of the two species, and to retain most of the variation as call properties were correlated, we performed a Principal Component Analysis (PCA) based on ten call variables extracted for 367 advertisement calls: 256 *U. systoma* calls extracted from 13 individuals and 111 *U. globulosus* calls extracted from seven individuals. We used the scree plot function in RStudio Version 4.0.3 [59] to plot a line and determine the number of factors with eigenvalues values > 1. We also plotted the cumulative variance to assess the amount of explained variance and we prepared a scatterplot to graphically represent the relationship between the two species from the two principal components retained. Variables were selected if loading into PC > 0.76. To test for significant differences between the two PCs retained for the analysis, we performed a one-way ANOVA on PC1 and PC2 with species as dependent variables and PCs as independent variables. We performed this analysis to understand whether the variation in the call properties between two species was significantly different.

### Ecological requirements and habitat segregation

The environmental variables were selected depending on their impact of the call properties of the species, amphibians in general, and to answer our hypotheses. The variables we selected were also used by the literature to determine their relationship with the vocal behaviour of the target species [22–24, 51]. To determine the segregation in calling habitat between the two *Uperodon* species we ran a one-way ANOVA after checking the assumptions for the independence of variables for both target species i.e. air temperature, water temperature, relative humidity, windspeed, depth of waterbody, distance from an individual to the bank (divided by the size of water body). Our dataset was not normally distributed as residuals were against fitted values, so we used a Kruskal–Wallis H test by ranks to test for differences between the calling habitat of the two species. All the analyses were done in RStudio Version 4.0.3 [59].

## Supplementary Information

**Additional file 1.**

## Data Availability

The datasets supporting the conclusions of this article are available in the figshare repository (https://figshare.com/s/14d1fdb17f576acf2502). The call recordings used for analyses and result of this are article are available in the FonoZoo sound library (http://www.fonozoo.com/fnz_buscar.php) with accession number: 14171 —14194.

## Abbreviations

ANOVA: Analysis of variance
GPS: Global Positioning System
FTT: Fast Fourier Transformation
SVL: Snout-Vent Length
