# Supplementary material for: Coexisting good neighbours: acoustic and calling microhabitat niche partitioning in two elusive syntopic species of balloon frogs, Uperodon systoma and U. globulosus (Anura: Microhylidae) and potential of individual vocal signatures
Source: BMC Zool. 2022 May 27;7:27. doi: 10.1186/s40850-022-00132-x (PMC10127398; doi:10.1186/s40850-022-00132-x)
Supplement: Supplementary file 1 — Additional file 1. [file 40850_2022_132_MOESM1_ESM.docx]

Coexisting good neighbours: acoustic and calling microhabitat niche partitioning in two elusive syntopic species of Balloon Frogs, *Uperodon systoma* and *U. globulosus* ([Anura](https://amphibiansoftheworld.amnh.org/Amphibia/Anura): [Microhylidae](https://amphibiansoftheworld.amnh.org/Amphibia/Anura/Microhylidae)) and potential of individual vocal signatures.

Vishal Kumar Prasad, Ming-Feng Chuang, Abhijit Das, K. Ramesh, Yoonjung Yi, K. P. Dinesh and Amaël Borzée

Supplementary data: Table S1. The relationships of call properties with air temperature, body length, body mass, and body condition:

| Coefficient of correlation | Air temp | | SVL | | Body mass | | Body condition | |
| --- | --- | --- | --- | --- | --- | --- | --- | --- |
|  | *U. systoma* | *U. globulosus* | *U. systoma* | *U. globulosus* | *U. systoma* | *U. globulosus* | *U. systoma* | *U. globulosus* |
| Call rate | 0.259 | 0.316 | -0.061 | 0.086 | 0.274 | -0.626 | 0.451 | -0.647 |
| Call duration | 0.664 | -0.408 | -0.386 | 0.619 | -0.499 | 0.715 | -0.392 | 0.642 |
| Rise time | 0.456 | -0.389 | -0.379 | 0.630 | -0.526 | 0.707 | -0.437 | 0.631 |
| Fall time | 0.519 | -0.412 | -0.268 | 0.614 | -0.467 | 0.726 | -0.448 | 0.653 |
| Low freq (Hz) | 0.357 | 0.526 | -0.305 | -0.353 | 0.113 | -0.553 | 0.427 | -0.513 |
| High freq (Hz) | -0.203 | 0.016 | 0.037 | **0.822*** | 0.154 | 0.087 | 0.192 | -0.024 |
| Delta power (dB) | 0.466 | -0.802 | 0.472 | -0.304 | 0.234 | 0.594 | -0.068 | 0.644 |
| Frequency modulation of low freq | -0.285 | 0.233 | -0.092 | -0.350 | 0.365 | 0.048 | 0.610 | 0.096 |
| Frequency modulation of high freq | 0.371 | 0.267 | 0.612 | 0.263 | 0.448 | -0.470 | 0.123 | -0.513 |
| Dominant harmonic | -0.418 | -0.163 | 0.110 | 0.757 | 0.087 | 0.259 | 0.031 | 0.159 |

The values are shown separate for *Uperodon systoma* and *U. globulosus*. We highlighted the values with asterisk when the relationship is significant (p-value < 0.05).
